# MR-FPN: Multi-Level Residual Feature Pyramid Text Detection Network Based on Self-Attention Environment

**DOI:** 10.3390/s22093337

**Published:** 2022-04-27

**Authors:** Jianjun Kang, Mayire Ibrayim, Askar Hamdulla

**Affiliations:** College of Information Science and Engineering, Xinjiang University, Urumqi 830046, China; kangjianjun@stu.xju.edu.cn (J.K.); askar@xju.edu.cn (A.H.)

**Keywords:** text detection, attention, deep learning, feature pyramid

## Abstract

With humanity entering the age of intelligence, text detection technology has been gradually applied in the industry. However, text detection in a complex background is still a challenging problem for researchers to overcome. Most of the current algorithms are not robust enough to locate text regions, and the problem of the misdetection of adjacent text instances still exists. In order to solve the above problems, this paper proposes a multi-level residual feature pyramid network (MR-FPN) based on a self-attention environment, which can accurately separate adjacent text instances. Specifically, the framework uses ResNet50 as the backbone network, which is improved on the feature pyramid network (FPN). A self-attention module (SAM) is introduced to capture pixel-level relations, increase context connection, and obtain efficient features. At the same time, the multi-scale enhancement module (MEM) improves the expression ability of text information, extracting strong semantic information and integrating the multi-scale features generated by the feature pyramid. In addition, information regarding the upper features will cause loss when the feature pyramid is passed down step by step, and multi-level residuals can effectively solve this problem. The proposed model can effectively improve the fusion ability of the feature pyramid, provide more refined features for text detection, and improve the robustness of text detection. This model was evaluated on CTW1500, Total-Text, ICDAR2015, and MSRA-TD500 datasets of different kinds and achieved varying degrees of improvement. It is worth mentioning that the F-measure of 83.31% obtained by this paper on the Total-Text dataset exceeds that of the baseline system by 5%.

## 1. Introduction

At present, scene text detection and recognition technology, as a branch of computer vision, has become a topic that researchers focus on. Scene text detection mainly detects location information regarding text areas from pictures containing text for subsequent text recognition tasks. Nowadays, text detection and recognition technology can be found in many industrial fields [1,2], such as autonomous driving, license plate recognition, intelligent sorting technology for express delivery, real-time translation, etc. However, considering the stability and security of the system, traditional technologies were used in the past. In recent years, as deep learning has become a mainstream trend in research methods, many deep-learning-based text detection algorithms have been proposed and achieved advanced results in text detection datasets.

**Figure 1:**
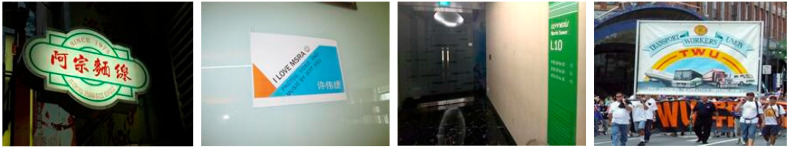
Different kinds of text instances in scene text, including straight text, horizontal text, curved text, and multilingual text.

As shown in Figure 1, scene text contains rich and diverse elements, which lead to huge challenges in the process of detection. The first challenge is that the shape, size, and color of scene texts are very different, and most of them are curved texts in real life. Secondly, foreground texts are usually similar to the background, which can easily cause misjudgment via machine detection. Finally, the image acquisition equipment, lighting, incomplete text information, and other problems will also have a great impact on the quality of the image.

Text detection algorithms mainly include traditional methods [3,4,5] and deep learning methods [6,7,8,9,10,11,12,13,14,15,16,17,18]. Traditional learning requires the elaborate design of features and is restricted by prior information, such as strokes and widths, and cannot process complex scene texts. Later, inspired by object detection [19,20,21] and instance segmentation [22], traditional artificial designed features were abandoned, and convolutional neural networks were used to extract features automatically. Deep learning methods are divided into regression-based algorithms and segmentation-based algorithms. Regression-based algorithms describe text instances through anchor boxes, but such algorithms are more inclined to straight text and are insensitive to curved text. The segmentation algorithm judges whether each pixel is a text target on the basis of pixel level and then is combined with a post-processing algorithm to integrate it into a text box. In fact, most segmentation algorithms [9,13,14] use reconstructed text instances, such as the use of the disc method, different kernels, etc. However, they all need to process labels. In general, they can detect text instances of any shape well, but it is difficult to distinguish adjacent texts with these algorithms, and the problem remains that the robustness is still not high.

The feature pyramid network (FPN) [23] has the ability to encode text information. By transferring high-level semantic features from top to bottom and connecting them horizontally, it gradually fuses them with shallow semantic features to obtain multi-scale features of the input image and finally splice features of different scales. This method bridges the gap of insufficient semantic information in the detection process and improves the accuracy of prediction and segmentation. Even so, there are still the following shortcomings: the top-level semantic information will be gradually lost in the process of high-level semantic features being passed down; simply restoring low-resolution images to high-resolution cannot capture context dependencies well; finally, the different feature layers are just simple splices of dimensions. Although they contain a large number of detailed features, the strong semantic information required for detection is insufficient.

In this study, we proposed a multi-level residual feature pyramid network in the context of self-attention, which aimed to enhance the ability of feature pyramids to express and aggregate textual information and improve the robustness of arbitrary-shaped text detection. Specifically, we applied multi-level residuals to compensate for the loss of semantic information during top-down propagation. In addition, we designed a self-attention module (SAM) that combined channel attention and spatial attention to enhance contextual connections and highlight the correct semantic feature area. Finally, the multi-scale enhancement module (MEM) extracted rich features in semantic information from both global and local levels and further fused with the output of the feature pyramid to obtain more detailed features. In summary, the contributions of this paper are summarized as follows:

- We proposed a multi-level residual model in the context of self-attention for the text detection of arbitrary shapes.
- A self-attention module was designed which can resolve the channel and space dependencies after up-sampling, enhance the contextual connection, adaptively focus on the main features, ignore the secondary features, and only introduce a small number of parameters.
- The multi-level residual network mainly compensated for the loss of upper-level features during top-down transmission. The multi-scale enhancement module considered the global and local complementarity and extracted highly semantic information well.
- In order to prove the superiority of the model, we conducted experiments on multiple benchmark datasets, including horizontal, multi-directional curved text, etc., and achieved good results. Among them, there were significant improvements in Total-Text and CTW 1500.

## 2. Related Work

As mentioned above, traditional learning requires tedious processing, while deep learning can automatically obtain detection results by only inputting pictures, which greatly simplifies the processing process. Firstly, this section introduces deep learning detectors in the current research area and then focuses on the common attention mechanisms in the image domain.

### 2.1. Regression-Based Algorithms

Object detection [24,25] is mainly divided into Faster-RCNN, YOLO, and SSD, etc. Inspired by the former, many regression-based algorithms use multiple convolution kernels of different scales to generate a large number of anchor boxes in the feature map and then use a simple non-maximum suppression algorithm [26,27] to filter further fine text boxes. Textboxes [28] improves on the SSD network by using a 1 × 5 convolutional kernel to increase the perceptual field while modifying the aspect ratio and the size of the default box (similar to the anchor box of Faster-RCNN), but is only able to cover horizontal text areas. Ma et al. [29] devoted themselves to solving the problem of multi-directional text and proposed an RRPN algorithm to generate rotating bounding boxes, introduced angle information, and proposed a Rotated Region of Interest (RRoI) pooling layer to combine arbitrary orientations; the proposal was projected into the feature map of a text region classifier. R2CNN [30] proposed a two-stage algorithm that uses an RPN proposal network to generate axis-aligned boundaries and employed pooling kernels of different sizes to capture textual features in multiple orientations for predicting textual and non-textual scores. However, the size of the traditional anchor frame was artificially set and could not completely match a single text instance. ContourNet [15] designed an Adaptive RPN module to generate candidate frames with higher accuracy for text instances. ABCNet [31] used a third-order Bezier curve to fit text regions for the first time, and arbitrary shape text detection took another big step forward.

### 2.2. Segmentation-Based Algorithms

Segmentation-based algorithms are used as a semantic segmentation problem, using fully convolutional networks (FCN) to segment text regions from the background. First, the score map is predicted by the network, and then the text pixels are grouped, the text mask is output, and finally, the text pixels are merged. In order to solve the arbitrariness of text shape, LYU et al. proposed the Mask TextSpotter [32] algorithm based on the Mask R-CNN framework, which segmented each character individually. Although the detection accuracy was improved, it lacked character-level annotation training sets, and manual labeling takes a lot of time. TextSnake [9] first proposed a disc representation that can completely cover each text instance, but the post-processing algorithm was complicated. FCENet [18] is a further improvement on TextSnake to model text instances in the Fourier domain. DBNet [33] proposed a differentiable module to solve the problem of gradient differentiability, and an adaptive threshold replaced the traditional fixed threshold, which improved the detection performance. PSENet [13] addressed the problem of adjacent text instances sticking together during detection. Text instances are surrounded by multiple kernels, and post-processing designs a progressive scale expansion network. In order to reduce the time cost and speed up the inference speed, PANet [14] proposed a new text pixel aggregation post-processing algorithm that improved detector inference time. Unlike most of the previous algorithms, TextFuseNet [16] implemented the detection task simultaneously using multiple branches, learning characters, words, and global features.

### 2.3. Attention Mechanism

The attention mechanism is used to allow a neural network to selectively focus on some important features, assign large weights to some informative features, and suppress redundant information. At present, the most widely used mechanisms in computer vision are channel attention and spatial attention. Spatial attention focuses on where the features that need attention are located, and channel attention specifically needs to pay attention to what features are meaningful. In 2017, Hu et al. [34] proposed a channel attention mechanism called SENet, which only uses two fully connected layers for processing and assigns different weights to the channels of certain feature maps. CBAM [35] uses both channel attention and spatial attention; spatial attention is computed using K × K 2D convolution and then combined with channel attention. Although it can increase the accuracy, it also produces a huge amount of computation. NLNet [36] adopts a self-attention mechanism to model pixel-level pairwise relationships.

## 3. Proposed Method

In this subsection, a more efficient and accurate scene text detection algorithm is obtained in order to accurately separate similar text instances. In this paper, the PSENet algorithm was used as the baseline network, and three improvements were made on the basis of the original algorithm. The proposed method did not delete the original model of the author, but mainly upgraded the feature pyramid network and further optimized the feature fusion network, aiming to obtain more refined multi-scale features. This section mainly describes the following aspects, including the mainframe of the algorithm, the multi-level residual feature pyramid (MR-FPN), the self-attention model (SAM), and the multi-scale feature enhancement module (MEM).

### 3.1. Overall Architecture

**Figure 2:**
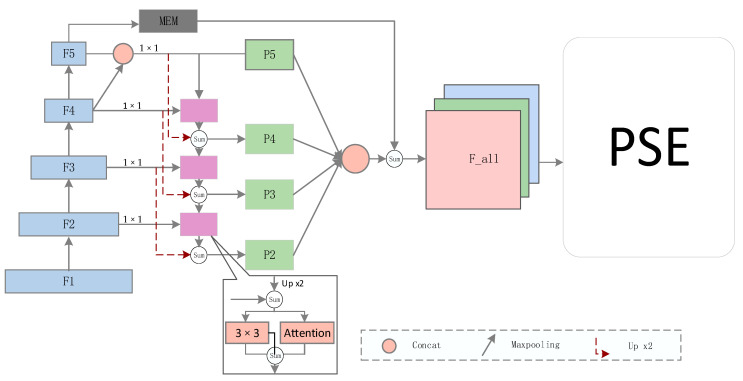
The overall structure of the network. The left side represents the feature extraction and fusion part, and the right side represents the progressive scale expansion algorithm. In addition, MEM stands for multi-scale enhancement module, and Attention stands for self-attention module. Multiple red dotted lines form a multi-level residual network.

The basic model improved in this paper is shown in Figure 2, which is mainly divided into three parts: the feature extraction network, the feature fusion network, and the progressive scale expansion network. In this paper, ResNet50 was used as the backbone network to extract basic texture features for the subsequent network, and the multi-residual feature pyramid network was used to fuse features of different scales to generate a more refined feature F_all for the segmentation task.

Finally, the feature F_all undergoes a 3 × 3 convolution and a series of 1 × 1 convolution operations to obtain the corresponding segmentation mask value, denoted as S1, S2, ..., Sn. For the reasoning part of the algorithm, it is assumed that n segmentation instances S1, S2, ..., and Sn are predicted, and the final complete shape is obtained using the progressive scale expansion algorithm. Generally, the search starts from the smallest text kernel S1, and then gradually expands the pixel point of the kernel. Searching from the smallest kernel removes the problem of detecting adjacent text as one text instance. Since this paper adopted the same inference algorithm as the baseline model, please refer to the PSENet [13] algorithm for more specific details.

### 3.2. Multi-Level Residual Feature Pyramid Network

The multi-level residual feature pyramid network is an improvement of the feature pyramid network (FPN), which is more conducive to the fusion of features and makes up for the loss of semantic information in the process of top-down transfer of features. First, ResNet50 generates multiple features of different scales, and the number of channels of each feature is {256, 256, 512, 1024, 2048}, which are, respectively, denoted as {F1, F2, F3, F4, F5}. Because each feature layer has different semantic information, the result of the maximum pooling layer in the feature layer of F4 is concatenated with the feature layer of F5 to increase the intermediate semantic feature information of F5. Each feature layer undergoes 1 × 1 convolution to reduce the channel dimension to 256 to ensure the consistency of the channel dimension in the subsequent fusion process. It is assumed that the obtained features are {C5, C4, C3, C2}. Generally speaking, from top to bottom, it is the combination of 3 × 3 and the attention mechanism after the fusion of C5 up-sampling and C4. In addition, in the process of the downward transmission of high-level semantic features, the closer to the bottom layer, the more serious the loss of upper-level feature information, so the multi-level residual can solve this problem very well. Upper-level feature up-sampling is sequentially added to the next-level output to obtain the final result, which is represented by {P4, P3, P2}, respectively. In fact, C5 is also equal to P5, thus forming a multi-level residual feature pyramid.

### 3.3. Self-Attention Module

**Figure 3:**
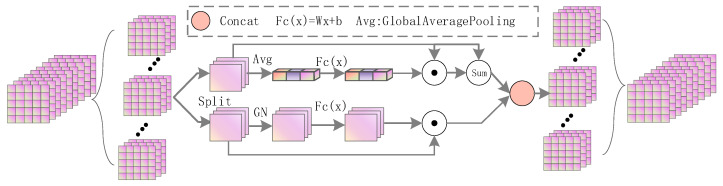
Structure of the self-attention module.

The self-attention module can enhance the semantic context connection, make up for the spatial loss caused by up-sampling, highlight the main features, and ignore redundant features. As shown in Figure 3, it includes channel attention and spatial attention, does not use any convolution, and has very few parameters. This module randomly groups the input feature maps according to the number of channels. For each sub-feature, one branch uses the channel relationship to generate a channel attention map, and the other branch uses the spatial relationship to generate a spatial attention map, then assigns weights to the original features, and then splices them by channel to form an attention unit, and all sub-features are re-aggregated into one feature. In fact, for a given feature, $X\in R^{\left(C\;\times \;H\;\times \;W\right)}$, C, *H*, and *W* represent the channel number, width, and height of the feature, respectively. First, the features are divided into M groups along the channel direction,$\;X\;=\left[X_{1},X_{2},\ldots ,{\;X}_{M}\right]$, $X\in R^{\left(C/M\;\times \;H\;\times \;W\right)}$. During the training process, weight coefficients are gradually generated for each sub-feature.

Specifically, each attention unit has two branches, supposing the segmented feature is $X_{k1},X_{k2}\in R^{\left(C/2M\;\times H\times W\right)}$. For the channel attention branch, in order to reduce the number of parameters, the convolution of SE is not used. First, global average pooling is used to obtain global information s, and then the assigned weights are obtained through a simple operation $X_{k1}^{\prime }$. In order to enhance the original features, they are fused with the attention features.

(1) $$s=F_{gp}(X_{k1})=\frac{1}{H\times W}\sum_{i=1}^{H}\sum_{j=1}^{W}X_{k1}(i,j),$$

(2) $$X_{k1}^{\prime }=\sigma (F_{c}(s))\cdot X_{k1}=\sigma (W_{1}s+b_{1})\cdot X_{k1},$$

On the other hand, the spatial attention branch is complementary to the channel attention, and the spatial statistics are first obtained using the group norm. Using FC() to enhance the representation of $X_{k1}^{\prime }$, the output of spatial attention is obtained as follows.

(3) $$X_{k1}^{\prime }=\sigma (W_{2}\cdot \mathrm{GN}(X_{k2})\;+b_{2})\cdot X_{k2},$$

### 3.4. Multi-Scale Enhancement Module

The multi-level residual feature pyramid gets features Pi of different scales, *i* = 2, 3, 4, 5. Pi is up-sampled to the same resolution as P2, splicing to a feature layer with a channel dimension of 1024. High-level semantic features are gradually diluted due to pyramids that go through convolution and sampling. In this paper, we designed a multi-scale enhancement module which makes full use of strongly semantic information and improves the feature representation ability, considering both global features and local features. Although F5 is not strong in detail features, it contains rich semantic information. Therefore, the F5 features of ResNet50 are directly passed through convolution kernels of different sizes.

**Figure 4:**
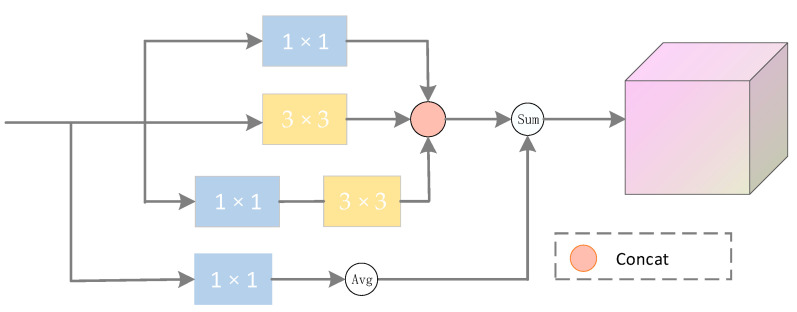
Multiscale augmentation module.

As shown in Figure 4, the feature extraction block consists of multiple Conv layers with different kernel sizes. When F5 features are input, 3 × 3 and 1 × 1 are used to adaptively extract local features. At the same time, 1 × 1 convolution also has the task of changing the number of channels. In addition, after the 1 × 1 convolution of the bottom branch reduces the channel dimension to 1024, the global average pooling layer captures the global features, and finally, the local features and the global features are fused to obtain more detailed features.

## 4. Experiments

This subsection introduces the public datasets required in the experiments and then conducts ablation experiments for each improved module to verify the effectiveness of the module. Finally, the scores of our algorithm and other advanced algorithms are compared.

### 4.1. Datasets

To fully validate the ability to detect arbitrary-shaped text, we evaluated the model on four challenging public datasets. The four benchmark datasets were ICDAR2015 [37], CTW 1500 [38], Total-Text [39], and MSRA-TD500 [40].

CTW1500 is a long-curved text training set, which is different from the ICDAR series of datasets. It uses polygonal annotation. Fourteen points can enclose a closed text area of any shape. The dataset has 1000 training images and 500 testing images.

ICDAR2015 has 1500 images, 1000 for training and 500 for testing. The size of the pictures is 1280 × 720, and most of them are composed of street view elements, including supermarkets, shopping malls, etc. Most of them are horizontal straight text. In addition to the high resolution, the text information of some images is blurred, the image background is more complex, and the text scale changes greatly. In each annotation file, each line represents the rectangular bounding box information of a text instance and represents the four points of coordinate information of the upper left, upper right, lower right, and lower left of the rectangle.

Total-Text mainly solves the detection and recognition of natural scene text in English. The dataset includes a total of 1550 pictures. The text is mainly horizontal, multi-directional, and curved. Because many objects and texts in natural scenes have similar textures, such as discs and fences, processing is often more difficult than that for linear texts.

MSRA-TD500 is a multilingual dataset with a total of 500 natural scene images (training 300 + test 200). The text in the pictures is clearly visible, and most appear on the guide cards. Dataset labels are usually in row units, and for hard-to-recognize text, text instances are often labeled as ‘difficult’. The resolution of each image is very high, considering the diversity of text and the complexity of the image background, so it is a relatively challenging dataset at present.

### 4.2. Implementation Details

All the experiments in this paper were carried out in the Linux environment, the programming compiler used python 3.6, and pytorch was used as the deep learning framework. We were equipped with an NVIDIA RTX 2080Ti GPU graphics card for this experiment. The initial learning rate was set to 0.001, the batch size was 4, and the stochastic gradient descent method was used as the optimization algorithm for training. The whole training was carried out for 600 epochs, and the learning rate decayed by 0.1 after every 200 training epochs. During the whole training process, we did not use any additional text detection training sets for pre-training, such as synthetic training sets, etc. We only used ResNet50 pre-trained on ImageNet as the backbone network to train from scratch.

In this training, all the text instances marked “Do Not Care” in the label file were not used. The balance factor of the loss function was 0.7. Considering the learning difficulty of positive and negative samples, we used Online Hard Example Mining (OHEM), and the ratio of positive and negative samples was set to 3. We augmented the training data. (1) Each image was randomly scaled by the ratio {0.7, 0.8, 0.9, 1.0, 1.1, 1.2, 1.3}. (2) Since the image size of each dataset was different, all input images were determined to be 640 × 640 before being sent to neural network training. (3) The images were randomly rotated within (−10°, 10°).

### 4.3. Ablation Studies

In order to fully verify the effectiveness of each module, we performed ablation experiments on the CTW1500 curved dataset. The ablation objects were the multi-scale enhancement module (MEM), self-attention module (SAM), and multi-level residual feature pyramid network (MR-FPN). In ablation experiments, we tested the addition of a self-attention module to the feature pyramid network (FPN), the addition of a multi-scale enhancement module to the feature pyramid network (FPN), and the final multi-level residual feature pyramid network, and a combination of various modules. The final experimental results are shown in Table 1.

As can be seen from Table 1, the precession, recall and F-measure of the baseline algorithm we selected on the CTW1500 dataset were 80.57%, 75.55%, and 78.0%. However, the method MR-FPN + SAM + MEM proposed in this paper achieved 83.83%, 77.71%, and 80.65% in terms of precession, recall and F-measure in the same dataset. Compared with the baseline, our method had a 2.65% higher F-measure, and the result was significantly better than the baseline’s algorithm without pre-training. Each ablation experiment is introduced separately below, and all used the same CTW1500 dataset and did not change other experimental parameters.

#### 4.3.1. MEM

Adding a multi-scale enhancement module to the FPN can extract the high semantic information generated by ResNet50, which was used to obtain more refined features. As can be seen from Table 1, the precision of the MEM module was improved by 5.01%, and the F-measure was improved by 0.76%. This experiment shows that the multi-scale enhancement module can extract semantic information of different scales for fusion.

#### 4.3.2. SAM

The self-attention mechanism can effectively suppress redundant feature information in complex backgrounds and highlight the main features. Therefore, more precise text bounding box coordinates can be obtained. Compared with the baseline, the precision, recall and F-measure gained 1.74%, 1.96% and 1.35%, respectively. The boost was better than the first improvement. It can better suppress backgrounds or objects similar to text and improve the precision.

#### 4.3.3. MEM + SAM

After considering a single module, we fully fused the two modules in one network to obtain richer semantic information. The experiments show that our fusion was effective, and the detection effect was significantly improved. Specifically, our F-measure improved by 2.43% compared to the baseline. Compared with the single MEM and SAM modules, the F-measure increased by 1.67% and 1.08%, respectively.

#### 4.3.4. MR-FPN

The multi-level residual feature pyramid network can make up for the loss of features and fully extract text area information. Text instances can be completely described, even for the text of arbitrary shape. It can be seen from Table 1 that the precision, recall, and F-measure of our network achieved excellent results of 82.53%, 77.93%, and 80.17%, of which the F-measure was higher than the baseline’s by 2.17%, and the detection effect was obvious and outperformed PSENet.

### 4.4. Evaluation on Benchmark Datasets

To demonstrate the state of the art of our method, we compared it, the baseline, and other efficient detection algorithms on multiple different datasets, all achieving varying degrees of improvement. In addition, unlike the baseline, we also trained on the MSRA-TD500 multilingual dataset, which is a dataset not covered by the baseline, and achieved unexpected results. By comparing the performance of P, R, and F on the four datasets, the results show that our network had efficient detection ability and certain robustness.

#### 4.4.1. Detecting Curve Text

To test the model’s performance on curved text, we evaluated our algorithm on the Total-Text and CTW1500 datasets. The evaluation results of Total-Text and CTW1500 are presented in Table 2 and Table 3, respectively. During testing, we resized the images to 640 × 640.

As can be seen from Table 2, on the Total-Text dataset, without pre-training, the precision, recall, and F-measure of the baseline were 81.77%, 75.11%, and 78.3%, while our method achieved 88.17%, 78.96%, and 83.31%, respectively. The F-measure was improved by 5.01%, and the precision was improved by 6.4%, obtaining unexpected results. Compared to other state-of-the-art methods, the F-measure achieved by our method was 4.81% higher than TextSnake and 2.61% higher than the fastest DBNet algorithm.

At the same time, the algorithm proposed in this paper was also evaluated on the CTW1500 dataset. Unlike Total-text, this dataset is mostly long curved text. In Table 3, it is shown that we improved the F-measure by 2.65% compared to the baseline model, which is a significant improvement. Compared with other advanced algorithms, our algorithm is still among the advanced algorithms.

**Figure 5:**
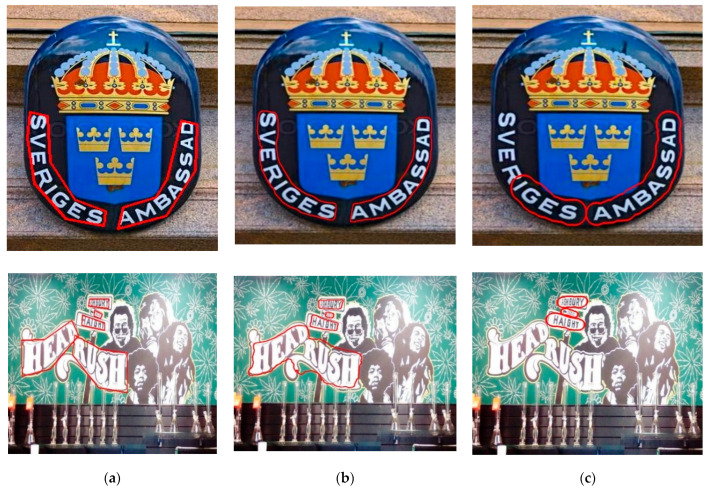
Visualization results on the Total-Text dataset, where (**a**) is the visualization result of ground truth, (**b**) is the result detected by the algorithm in this paper, and (**c**) is the visualization result of the TextSnake model.

**Figure 6:**
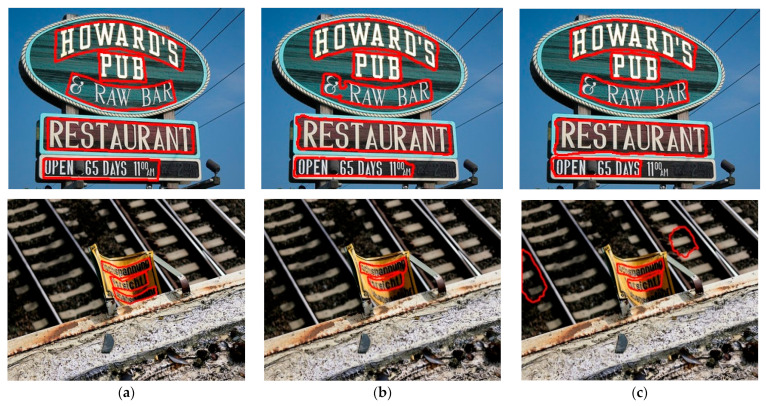
Visualization results on CTW1500, (**a**) for ground truth, (**b**) for the results of this paper’s algorithm, (**c**) for PSENet model detection.

The visualization results on the two datasets are shown in Figure 5 and Figure 6. Figure 5 shows the detection results using the TextSnake model and the model proposed in this paper on the Total-Text dataset. It can be seen that the model in this paper can correctly detect both artistic text and curved text, while the TextSnake algorithm can only detect part of the text or directly fails to detect the text. On the other hand, for curved text, the algorithm using progressive scale expansion is superior to the disc algorithm. The detection results of the baseline model algorithm and the text algorithm are given in Figure 6. It is obvious that the baseline model had missed and false detections, as shown in Figure 6c, while the algorithm in this paper eliminated this situation.

The experiments on the above two datasets show that the algorithm in this paper can handle curved text instances very well. Compared with other advanced algorithms, all the F-measure values displayed different improvements. In fact, by observing the text features of the pictures in the dataset, the text instances in the dataset are very clear, and there are only a few blurry pictures, most of which are shot at close range, so the detection accuracy is improved.

#### 4.4.2. Detecting Multi-Oriented Text

Pictures in real life include not only curved text, but also straight text in multiple directions. This paper evaluated our model on the ICDAR2015 dataset. It can be seen from Table 4 that the F-measure of this paper was improved by 0.3% compared with the baseline. Although the improvement was not high compared to the baseline, the model still has the ability to detect multiple directions.

**Figure 7:**
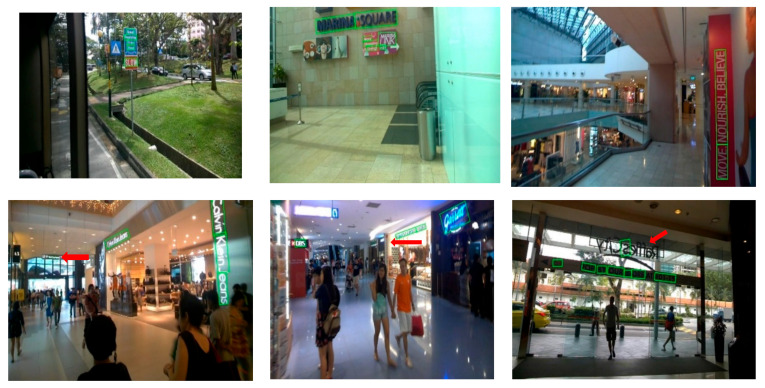
Some visualization results on the ICDAR2015 dataset; the arrows marked in red in the picture represent the text instances that were not detected.

As can be seen in Figure 7, a large number of text instances appear in the street and shopping malls, but these text instances are surrounded by complex background areas. Some undetected texts appear in the picture, such as blurred texts, texts in dark environments, and texts affected by multiple factors of illumination and distance, and it is difficult to detect them using the algorithm in this paper. The main reason for this is that the resolution of the ICDAR2015 dataset is high. If it is directly compressed to 640 × 640, it will cause text deformation. In addition, there are a lot of blurry pictures in the dataset, which interferes with text detection, most of the photographers shoot from a distance, and the picture quality is very poor. The environment has a high impact on this dataset, so the detection effect is not significantly improved.

#### 4.4.3. Detecting Multi-Language Text

In our experiments, we did not test the baseline method on the MSRA-TD5000 dataset, but in order to fully demonstrate the effectiveness of the model, we compared it with other advanced models. MSRA-TD500 is currently a very challenging dataset, and we used the image size of 640 × 640 for evaluation experiments. Table 5 shows the comparison results between the algorithm presented in this paper and other advanced algorithms. It can be seen that the F-measure of this method obtained 78.24%, which is basically the same as the TextSnake and DeepReg algorithms.

**Figure 8:**
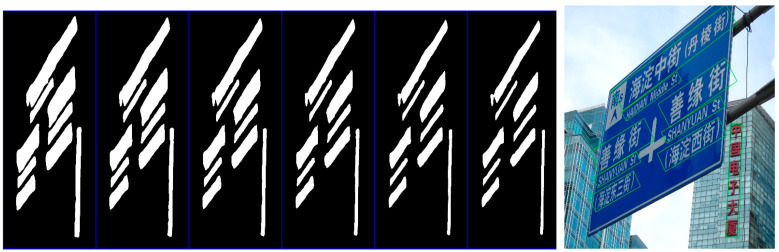
Visualization results of the binarization and multilingual text detection with different kernels predicted on the MSRA-TD500 dataset.

The binarization plot can be clearly seen in Figure 8, where the size of the kernel increases gradually from right to left, and the smallest kernel is able to predict the adjacent text instances better. On the multilingual dataset, this algorithm is able to accurately circle the text region of vertical text in a horizontal row of text.

## 5. Conclusions

Aiming to localize text regions in natural scene texts, this paper further optimized the fusion part of the PSENet algorithm and proposed a multi-level residual feature pyramid network in a self-attention environment, which can not only efficiently separate adjacent text instances, but also improves the detection effectiveness. After the previous experimental demonstration, each module plays an indispensable role. Although this paper did not use a pre-trained model, the results are still better than most algorithms. Finally, it can be concluded that the algorithm proposed in this paper can achieve good results on a variety of datasets, including long text, curved text, straight text, and multilingual text, and compared with the classic PSENet algorithm and the current popular detection algorithms, the detection performance was significantly improved.

In the future, there is still much room for improvement of the algorithm, and we are considering further optimization of the following aspects. For example:

➢ Most of the images in the ICDAR2015 dataset are blurred and heavily influenced by the environment, but the images in this dataset are also the most common in real life. Since some of the text is obtained under extreme conditions, such as unclear text under bright light exposure (sunlight or lights) or in dark environments, which seriously affects detection, consideration should be given in the future to how to eliminate the effect of this factor.
➢ Our algorithm has good robustness in close detection, but for distant text, our model still has some weaknesses. This is because when text is far away from the photographer, the text area becomes smaller, the text is blurred, the expressiveness of text features is weaker, and the extraction of features is not sufficient. Therefore, the enhancement of the feature extraction ability of text instances for small targets will be considered in the follow-up. In addition, if an image is compressed again, the text will be distorted, and some better algorithms will be used to compress images in the future.
➢ This paper increased the depth of the model to a certain extent, so it increased the consumption of time, and the model needs to be further optimized in the future.

## Figures and Tables

**Table 1:** Results under different modules; “P”, “R”, and “F”, respectively. represent precision, recall, and F-measure.

Backbone	MEM	SAM	MR-FPN	P	R	F
ResNet-50				80.57	75.55	78.0
ResNet-50	√			85.58	72.95	78.76
ResNet-50		√		82.31	77.51	79.35
ResNet-50	√	√		83.76	77.35	80.43
ResNet-50			√	82.53	77.93	80.17
ResNet-50	√	√	√	83.83	77.71	80.65

**Table 2:** The single-scale results on Total-Text. “P”, “R”, and “F” represent the precision, recall, and F-measure, respectively. The best results of all measures are bold.

Total-Text			
Method	P	R	F
LOMO [41]	75.7	**88.6**	81.6
TextDragon [42]	74.2	84.5	79.0
DBNet [33]	**89.3**	73.8	80.7
TextSnake [9]	82.7	74.5	78.4
SegLink++ [12]	80.9	82.1	81.5
TextField [43]	81.2	79.9	80.6
PSENet-1s [13]	81.77	75.11	78.3
Our-Method	88.17	78.96	**83.31**

**Table 3:** The single-scale results on CTW1500. “P”, “R”, and “F” represent the precision, recall, and F-measure, respectively. The best results of all measures are bold.

CTW1500			
Method	P	R	F
CTPN [44]	60.4	53.8	56.9
SAEmbed [45]	77.8	**82.7**	80.1
TextSnake [9]	**85.3**	67.9	75.6
CTD + TLOC [38]	69.8	77.4	73.4
TextDragon [42]	81.0	79.5	80.2
SEMPANet [46]	84.08	72.82	78.04
PSENet-1s [13]	80.57	75.55	78.0
Our-Method	83.83	77.71	**80.65**

**Table 4:** The single-scale results on ICDAR2015. “P”, “R”, and “F” represent the precision, recall, and F-measure, respectively. The best results of all measures are bold.

ICDAR2015			
Method	P	R	F
CTPN [44]	74.22	51.56	60.85
SegLink [47]	73.1	76.8	75
SSTD [48]	80.23	73.86	76.91
WordSup [49]	79.33	77.03	78.16
EAST [6]	83.57	73.47	78.2
RRPN [29]	82	73	77
DeepReg [50]	82	**80**	81
Lyu et al. [51]	**94.1**	70.7	80.7
PSENet-1s [13]	81.49	79.68	80.57
Our-Method	86.14	76.31	**80.92**

**Table 5:** The single-scale results on MSRA-TD500. “P”, “R”, and “F” represent the precision, recall, and F-measure, respectively. The best results of all measures are bold.

MSRA-TD500			
Method	P	R	F
EAST [6]	67.43	**87.28**	76.08
DeepReg [50]	77.0	70.0	74.0
Pixelink [7]	73.2	83	77.8
MOST [17]	81.2	74.8	77.9
TextSnake [9]	**83.2**	73.9	78.3
RRPN [29]	71.8	67	69.3
RRD [8]	73	87	**79**
Our-Method	80.87	75.77	78.24

## Data Availability

The public datasets in the experiments can be obtained: Total-Text: https://github.com/cs-chan/Total-Text-Dataset (accessed on 18 December 2021). CTW1500: https://ctwdataset.github.io/ (accessed on 18 December 2021). ICDAR2015: https://rrc.cvc.uab.es/?ch=4&com=downloads (accessed on 18 December 2021). MSRA-TD500: http://www.iapr-tc11.org/mediawiki/index.php/MSRA_Text_Detection_500_Database_%28MSRA-TD500%29 (accessed on 18 December 2021).
